# Study Protocol: Pegasus: psychotherapy incorporating horses for ‘therapy-resistant’ adolescents with autism spectrum disorders, a study with series of randomised, baseline controlled n-of-1 trials

**DOI:** 10.1186/s12888-024-05879-w

**Published:** 2024-07-10

**Authors:** Jenny C. den Boer, Helen Klip, Anita Blonk, Monique Lenselink, Shireen P.T. Kaijdoe, Melanie Tielkes, Amber van Zandbeek, Gerdine Bres, Mandy Herinx, Wouter G. Staal, Nanda Rommelse

**Affiliations:** 1 Karakter, Child and Adolescent Psychiatry, Ede, the Netherlands; 2 Karakter, Child and Adolescent Psychiatry, Nijmegen, the Netherlands; 3 Department of Psychiatry, Radboud University Medical Centre, Nijmegen, the Netherlands; 4 Gagelhoeve, Mill, the Netherlands; 5 Paardenblik, Wageningen, the Netherlands; 6 Horses en Co, Heerjansdam, the Netherlands; 7 Institute for Brain and Cognition, Leiden, the Netherlands; 8 Department of Developmental Psychology, Utrecht University, Utrecht, the Netherlands

**Keywords:** Autism, Emotion dysregulation, Therapy-resistant, Adolescent, Psychotherapy incorporating horses (PIH)

## Abstract

**Background:**

For people with autism spectrum disorder (ASD), daily life can be highly stressful with many unpredictable events that can evoke emotion dysregulation (ED): a strong difficulty with appropriately negative affect regulation. For some of the patients with ASD, treatment as usual does not prove to be effective for ED. They may be at risk of life-long impairment, development of other disorders and loss of motivation for most regular forms of therapy. A highly promising method that may prove effective for therapy-resistant individuals with ASD is Psychotherapy incorporating horses (PIH). PIH uses the interactions of the horse and the patients on the ground and does not include horseriding. While often met with prejudgment and scepticism, reports from parents and therapists as well as a recent systematic review suggest that PIH may have beneficial effects on youths with ASD. Therefore, we examine clinical outcomes both in the short and in the long terms of PIH offered to adolescents with ASD and severe ED despite regular therapy.

**Methods:**

A total of 35 adolescents aged 11–18 years with ASD will receive PIH during 15 sessions once a week with randomization to five different groups differentiating in baseline phase from 2 to 6 weeks. PIH uses horses to promote social awareness and self-awareness as well as relationship management and self-management. The primary outcome is the response to treatment on the Emotion Dysregulation Index (EDI). The secondary outcome measures include ASD symptom severity, quality of life, self-esteem, global and family functioning, and goal attainment. Assessments take place at the baseline (T0), at the end of baseline phase A (T1), after completion of intervention phase B (T2), after the end of post-measurement phase C (T3) and after one year (T4). Qualitative interviews of participants, parents and therapists will be held to reveal facilitators and barriers of PIH and a cost-effectiveness study will be performed.

**Discussion:**

This study aims at contributing to clinical practice for adolescents with ASD and persistent emotion regulation problems despite 1.5 year of treatment by offering Psychotherapy incorporating horses in a study with series of randomised, baseline controlled n-of-1 trials.

**Trial registration:**

www.ClinicalTrials.gov NCT05200351, December 10th 2021.

## Introduction

Autism spectrum disorder (ASD) is a neurodevelopmental disorder with an estimated worldwide prevalence of 1–3% [1]. Autism is marked by two phenomena. Firstly, patients with autism experience difficulties with integrating information. This may result in problems with understanding others (theory of mind), a more detail-focused style of processing information (executive functioning) or difficulties with imagining future events. Secondly, patients with autism have trouble with processing sensory information, which may result in either overwhelming or decreased experiences of sensory input. In severe cases, daily life can be experienced as chronically stressful and even traumatic. Even minor inconsistencies may evoke sudden and intense negative affective responses, also known as emotion dysregulation (ED) [2, 3]. ED increases the chance of rejection by others, thereby negatively impacting self-esteem [4] and increasing the chance of the development of comorbid internalizing and externalizing disorders [5, 6]. Moreover, ED may negatively influence socio-cognitive functioning in people with ASD as it increases the likelihood of misinterpretation of social cues towards more hostile perception bias with other people [7]. As such, ASD with severe levels of ED can be considered to have an ultra-high risk profile for developing other disorders, such as psychosis, anxiety, depression and eating disorders. Treatment targeting this maladaptive cycle may improve outcomes for individuals with ASD.

### ‘Therapy-resistant’ ASD

Care as usual (CAU) following typical guidelines may include psycho-education, medication, cognitive behaviour therapy and environmental adaptations. However, these do not diminish ED in some patients with ASD and there are currently no guidelines or treatment options for this subgroup of severely impaired, therapy-resistant individuals with ASD. The ‘dooming’ perspective of lifelong impairment may significantly demoralize patients and parents and increase the risk of creating a self-fulfilling prophecy with a cascade toward comorbid disorders and school dropout [8, 9].

### Adolescence: a (perhaps last? ) ‘Window of opportunity’ to intervene

In ASD literature, early childhood is considered to be the first ‘window of opportunity’ to intervene [10, 11]. During this period, significant treatment-related improvements in the core features of ASD have been reported [12]. We argue that a second (and perhaps last? ) ‘window of opportunity’ for intervention is adolescence for two reasons. Firstly, like early childhood, the adolescent age is related to major genetically pre-programmed neurological changes with a high sensitivity to environmental input across species [13, 14]. In humans, myelination and synaptic pruning in the prefrontal cortex increase sharply and neural connections between the prefrontal cortex and other regions of the brain are strengthened. This improves the efficiency of processing of information, controlling impulses, planning ahead, enhanced decision-making, processing of emotional experiences and social information, and also helps to more accurately evaluate rewards and risks [15]. Secondly, recently published longitudinal studies show that significant ASD symptom changes may occur in particular during adolescence [16, 17]. Offering intervention during this second ‘window of opportunity’ may therefore be the last opportunity to improve adulthood outcomes.

### A promising novel treatment method targeting emotion dysregulation: psychotherapy incorporating horses

Although often met with prejudgment and scepticism, reports from parents and therapists as well as systematic reviews suggest that Psychotherapy incorporating horses (PIH) may have beneficial effects on youths with ASD [18, 19]. Horses possibly offer additional value in psychotherapy: they are experts in nonverbal communication, do not judge, offer unconditional acceptance, provide tactile comfort and may give multiple real opportunities for learning new behaviours. However, due to the heterogeneity of the different interventions with horses and research methodology, evidence for the effectiveness of PIH on youths with ASD is currently insufficient to include PIH in the ASD treatment guidelines [20]. Just recently, a first step to reduce the heterogeneity is made by a consensus document recommending optimum terminology to describe interventions with horses to enhance scientific research [21]. The formerly used term equine-assisted therapy (EAT) is replaced by psychotherapy incorporating horses (PIH). Only licensed therapy professionals working in their particular disciplines who have received specialized training focused on incorporating interactions with horses into the therapy can perform PIH. They use individualized treatment plans to achieve the established goals. For this they have various treatment options at their disposal and they know when and how to incorporate horses.

In order to provide treatment perspective for (caretakers of) adolescents with ASD and treatment-resistant ED, an expert group developed a protocolled 15-weeks PIH, the Pegasus protocol.

### Rationale of the PIH-Pegasus

The objective of PIH-Pegasus is to improve emotion regulation in adolescents with ASD by strengthening their mentalization abilities and parental insight. Improving mentalization can be considered as the core of all treatment models [22]. PIH-Pegasus combines the most important elements of PIH and mentalization-based therapy, being a focus on currently felt affect, careful adjustment to the patient’s level of mentalization and arousal, a ‘not-knowing’ therapeutic stance and unassuming interventions [23, 24]. In addition, family sessions are included based on the most important elements of system therapy in families with a member with autism: to repair and improve relationships and cooperation and to create a context of hope and empowerment [25].

**Fig. 1:**
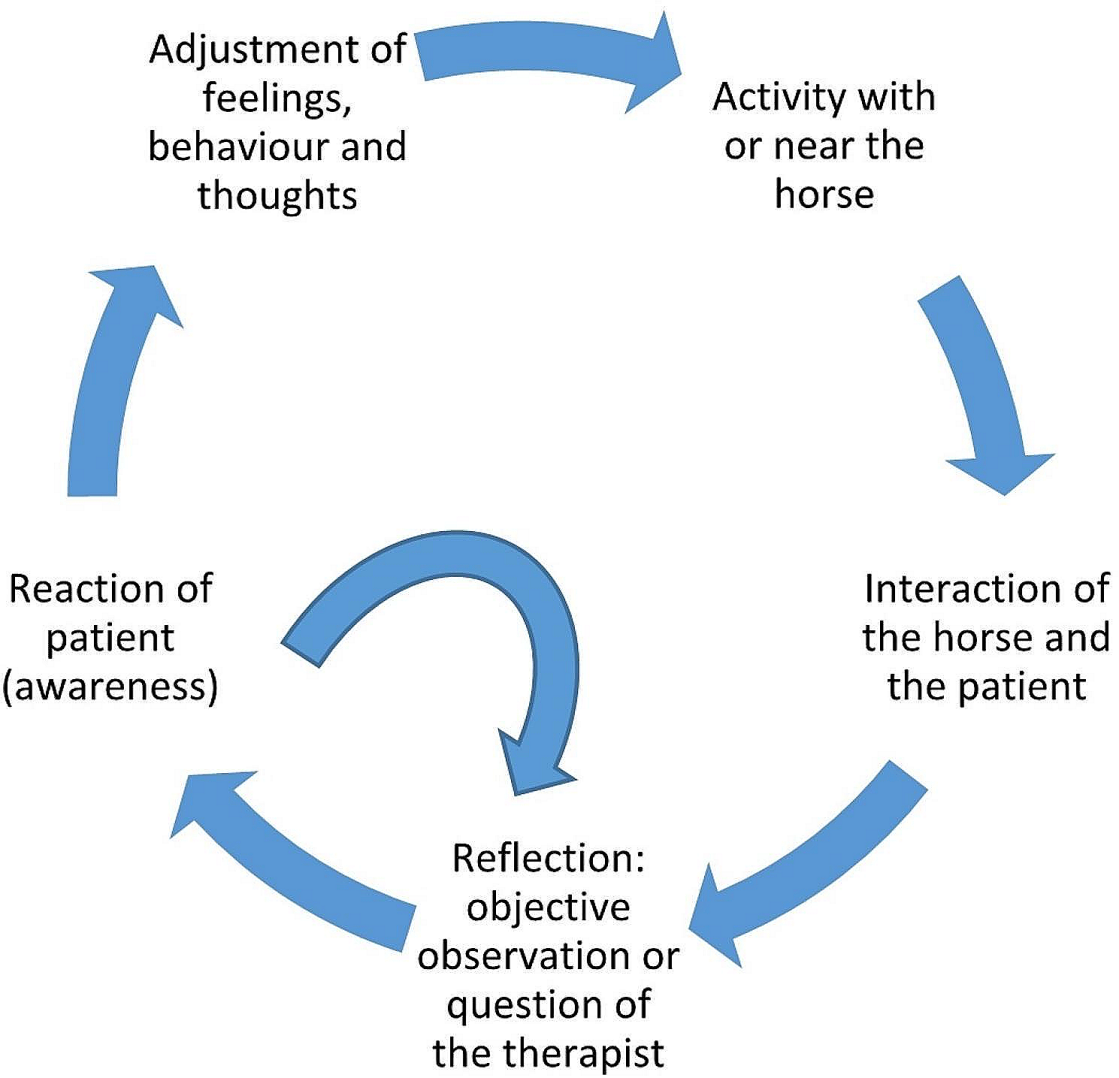
The PIH intervention cycle that will be followed several times in one session. Example using the PIH intervention (Fig. 1). A patient observes the horses (activity with or near the horse). One horse stands at some distance from the herd. The patient says the horse feels sad (interaction between horse and patient). The therapist asks why the horse feels sad (objective observation or question of the therapist). The patient thinks the other horses do not like it and do not want to stand next to the horse (reaction of the patient, in this case projection). The therapist asks if the patient recognizes this situation in his daily life (objective observation or question of the therapist). The patient starts talking about his situation in the classroom (reaction of the patient). The therapist asks the patient how he feels about the situation (objective observation or question of the therapist). The patient answers (reaction of the patient) and is asked to repeat the activity or to do a new one. The experiences and reflections can make the patient conscious of his own behaviour/feelings/thoughts and then he can adapt them. In this way patients are encouraged to find (their own) solutions

Only licensed professional mental health therapists with additional education to become an equine-assisted therapist are trained in the protocol. They only provide objective observations and are not ‘all-knowing persons’ who know the solution or what the horse’s intentions are. The therapist has an open, regulated attitude and leaves space for silence. The words of the participants are used to prevent interpretations of the therapist [26]. Therapists use the projections on the horse revealing the patient’s inner world, making this accessible to change. Most time will be spent on experiencing, 80% of the session on average. Reflecting by giving only objective observations of the horse and the patient will take 15% of the time and asking questions will take 5% of the time. This is called the 80-15-5 rule. The therapeutic process is shown in Fig. 1.

Unlike most other therapies with horses, only unmounted activities with or in the presence of a horse are used [18, 27]. Doing only unmounted activities gives the horse the freedom to move or not and makes sure that all his expressions are visible. The horse is used as a metaphor or model in order to provide opportunities to experience and practice new behaviours, including the expression of emotions. PIH does not seem to have any negative effect on the well-being of horses, as indicated by unchanged plasma cortisol levels and heart rate variability [28].

## Intervention

The Pegasus protocol consists of fifteen sessions, including twelve individual sessions and three system sessions with caretakers and the adolescent. If necessary to promote further transfer, five booster sessions can be scheduled after T3. Based on clinical experience and balancing between impact and burden for parents and participants, PIH sessions are held once a week (with the exception of holidays/illness) and last sixty minutes. As structure enhances safety and predictability for people with ASD, each session includes the following topics: assess well-being, discuss the transfer exercise, exercise with the horse, reflection moment, exercise with the horse, reflection moment, introduce the transfer exercise and evaluate the session. Three sessions with caretakers are scheduled, being the sixth, tenth and last session, to promote transfer and targeting dysfunctional patterns in the family. To ensure safety, a second qualified mental health and equine-assisted co-therapist is present during the scheduled system sessions.

All sessions follow the same structure and all therapists exhibit a consistent attitude of non-interpretation while employing identical techniques in order to ensure uniformity in the working elements.

When necessary, the protocol will be adjusted based on knowledge of the patient’s vulnerabilities. If the patient experiences a lot of stress, a familiar or easier exercise can be scheduled, so the order of the exercises can be adjusted when required. Adjustments are recorded in the therapy logbook after each session. The logbook will be used to improve the protocol in the future, if necessary.

### Therapists and horses

All therapists are BIG (Beroepen in de Individuele Gezondheidszorg) /SKJ (Dutch independent register for youth professionals) registered healthcare professionals and qualified to work with horses. To promote fidelity, they all had the same training. Additionally, use is made of intervision, fidelity scoring forms and logbooks. All therapists have had the same training of at least two years at the Dutch training centre for Equine assisted coaching & therapy Keulseweg. Intervision will take place with all therapists involved on a monthly basis. In order to maintain treatment fidelity, a consensus meeting will be organized every six months to be attended by all therapists. Treatment fidelity will also be assessed twice a year using a 7-item standardized observation instrument, the Pegasus Fidelity Scale. The scale is designed for this project after qualitative research by interviewing an expert panel, the recordings of which were transcribed verbatim, coded and analysed. Items are an open and not-knowing attitude, objective observations of the horse, objective observations of the patient, opportunities of experimenting, attention to transfer and safety of the horse, patients, materials and therapists.

All kinds of horses are used (Shetland, Icelandic, Friesian, KWPN, BWPN, Welsh and Fjord horses). All horses are at least five years old. The horses do not need any special training, but they must be mentally stable and used to handling by human beings.

### Emotion regulation, mentalization and communication in the sessions

During the sessions we address different aspects of mentalization (social awareness, self-awareness, relationship management and self-management) and communication. By improving mentalization and communication, the understanding of the intentions and behaviour of other people improve while lessening the emotion dysregulation and vice versa. We start with being aware of different aspects of the exterior of the horse and the behaviour of the horse (social awareness), followed by interacting with the horse and learning to respond to its behaviour (relationship management), shifting gently to the patient’s own behaviour, thoughts and emotions (self-awareness). In the last sessions self-management is more central (Table 1).

For patients we use more common terms to describe the focus of the sessions: the three O’s. During the first sessions, the focus is on discovering (in Dutch: Ontdekken) to enhance social awareness, (What is happening with the horse? What body signals are sent by the horse? ), relationship management (How do I react to the horse? How does it respond to me? ) and self-awareness (What kind of thoughts, feelings, emotions and behaviour do arise? What do I want to change? ). During the second phase of the process, problem solving by experimenting (in Dutch: Oplossen) becomes more important: behaviour shifts from unconscious to conscious and self-management improves. In this phase participants can try to find as many solutions to one situation as possible and experiment with these until they find the solution that works. During the last sessions, the focus is on consolidating (in Dutch: Onthouden). In practice all sessions have elements of all phases but with a different focus. The system sessions follow the same focus: first discover family patterns, to be followed by solving interaction problems and in the last session remembering what works best. They also have the same structure as the individual sessions, but with one addition: the progression of the objectives is measured during the system sessions with the Goal Attainment Scale (GAS) as part of the intervention. The system session contains exercises that promote working together, for example leading the horse through a ‘river with obstacles’. The therapists make objective observations and ask questions to both patient and parents, while also addressing the parents’ behaviour and making them aware of interactions. By repeating the exercises, the whole family can experiment with new behaviours.

As the heterogeneity of individuals with ASD asks for individualized treatment, the order of the activities (Table 1) can be adjusted when required. The first version of the protocol was tested on patients with ASS aged 11–18 years. Minor revisions were made after the first cases. We added the exercise of the comfort circle, which was easier to understand than the emotion thermometer.

### Assess personal goals

During the first sessions of PIH, the personal goals of the adolescent are determined using the Goal Attainment Scale (*see “Outcomes of the intervention - Goal Attainment Scale (GAS))”.* The goals of the adolescent may differ from the goals of the parents. This is an essential part of the therapy, as most of the adolescents have been sent to previous therapies with goals set by adults. The therapist can help the patient to set personal goals by means of observations made and by asking if the patient recognizes what happens during the session with respect to real life. Examples of personal goals verbalized by adolescents are: “How can I get friends and keep them?”, “I want to lessen my self-harm” or “I want to join the army, so how can I control my anger?” These goals are made measurable by dividing them in smaller steps so as to make them suitable for use in the GAS.

**Table 1:** Session topics, goals (main ER and mentalization element) and examples of activities

No	Topic	Goal (main ER and mentalization element)	Example of activities during session
1	Introduction	• Patient and therapist make contact with each other and the surroundings (SoA) • Structure of sessions is explained • First contact with the horse (SoA) • Learn to observe more carefully (SoA)	Observe the horse and make contact
2	Explore goals	• Possible goals for treatment are explored (SeA) • Practise in observing the horse (SoA) • Become aware of reactions of the horse (SoA) • Learn to relax (SM)	Groom the horse and so make physical contact
3	Set goals	• Goals of the therapy are made visible by using the GAS with pylons (SoA) • Learn to set realistic goals and divide these into smaller steps (SM) • Practise in making physical contact with the horse and observe the reactions of the horse (SoA) • Observe the interactions with the horse (SoA) • Cooperate with another being (RM) • Learn to operate in a new and sometimes frustrating situation (SM)	Halter the horse and lead the horse
4	Identify the social network	• All people relevant to the patient are made visible giving insight in who are important for the patient ((SoA) • Make visible whom the patient can address for help (RM)	Who is in your herd? (persons are symbolized by hoops and the roles they play are made conscious)
5	Promote cooperation	• Enhance creativity (SM) • Promote cooperation with another being (RM) • Observe the reactions of the horse (SoA) • Observe the patient’s own reactions (SeA) • Give a better understanding of the coherence between thoughts and behaviour ((SeA)	Horse in stable (a stable is made and the horse is put into the stable)
6	System session	• Get insight into the different family patterns with a focus on emotion regulation (SoA) • Evaluate the improvements made by assessing the GAS (SeA) • Enhance cooperation between all family members and the horse (RM) • Enhance objective observation (SoA) • Link behaviour and emotions (SeA) • Enhance taking care of each other and the horse (RM)	The river (a river with obstacles has to be crossed with the horse)
7	Change emotions	• Find out how emotions work (SeA) • Improve recognition of emotions (SeA) • Link emotions with behaviour (SeA) • Learn how emotions come and go and change and are expressed by observing the horses (SoA)	Hoops (hoops symbolize different emotions; how do they interrelate? )
8	Intensity of emotions differ	• Recognize own emotions, behaviour and thoughts (SeA) • Learn how emotions come and go and change and are expressed by the horse and the patient (SoA) • Improve self-consciousness (SeA)	Emotion thermometer or comfort circle (which behaviour/feelings/thoughts belong to low/medium or high stress? Deal with stress in learning situations)
9	Clear communications	• Interact with the horse in a clear way (RM) • Observe the reaction of the horse and respond in an adjusted way (RM) • Observe own reaction and link this to daily situations (SeA)	Build a slalom and lead the horse
10	System session	• Evaluate the improvements made by assessing the GAS (SeA) • Enhance positive interactions and cooperation in the family (SoA) • Give feedback in a positive and acceptable way (RM) • Give and receive leadership (RM)	Brains and hands (lead the horse while one person cannot use his hands, the other cannot talk, which role do you prefer? )
11	Deal with obstacles	• Set realistic goals and cooperate with the horse (RM) • Observe the behaviour and emotions of the horse ((SoA) • Deal with the emotions of the horse (RM) • Deal with a new and possible frustrating situation (SM)	Take an obstacle (the obstacle symbolizes an obstacle in real life)
12	Deal with a difficult situation	• Practise setting realistic goals and cooperate with the horse (RM) • Observe the behaviour and emotions of the horse (SoA) • Deal with the emotions of the horse (RM) • Deal with a new and possible frustrating situation (SM)	Horse on a sail (put the horse on a sail)
13	Deal with different personal traits	• Create better insight with the patient in his/her coping style (SeA) • Make use of his own resources (SM)	At the wheel (which traits belong to you and how do you act in different situations? )
14	Set boundaries	• Improve setting boundaries in an acceptable and firm way (RM) • Improve resilience (SM) • Use of nonverbal and verbal behaviour (SM)	Set your boundaries (prevent the horse from crossing your boundary)
15	System session	• Evaluate the improvements made by assessing the GAS (SeA) • Enhance positive interactions and cooperation in the family (RM) • Give feedback in a positive and acceptable way (RM) • Give and receive leadership (RM)	Build a trail with obstacles and lead the horse together through the trail

*Note * SoA = Social awareness, SeA = Self-awareness, RM = Relationship management, SM = Self-management

### Transfer

To promote transfer to daily life we use a transference leaflet with exercises. The generalization to daily life of skills learned in therapy can be maximized by elements of Cognitive Behaviour Therapy (CBT) that alter cognitive bias and maladaptive behaviour [29, 30]. The exercises promote the awareness of (the interconnection between) emotions, thoughts, physical sensations and behaviour, the role of triggers and the application of helping tools. In this way elements of cognitive behaviour therapy are combined with the experience-based principles of PIH. To enhance the transfer to real life, there are also the system sessions with caretakers and we encourage participants to talk about the session with their parents. In addition, if given permission, we take photos of special situations in the session and give these to the participants. At the end of the therapy the patient receives all his answers of the session evaluation forms in a frame together with some photos of remarkable situations of the session, thus offering a relapse prevention plan in a visible and attractive way.

## Aim of the Pegasus study

The primary objective of the Pegasus study is to examine the short-term (15 weeks) and long-term follow-up (1 year) effects of PIH-Pegasus in improving ED in adolescents with ASD and therapy-resistant ED. The secondary objectives are to examine the effects on goal attainment in the short and long terms, ASD symptoms, quality of life, self-esteem, family functioning and cost-effectiveness, as well as facilitators and barriers of the intervention.

## Methods

### Design of the Pegasus study

**Fig. 2:**
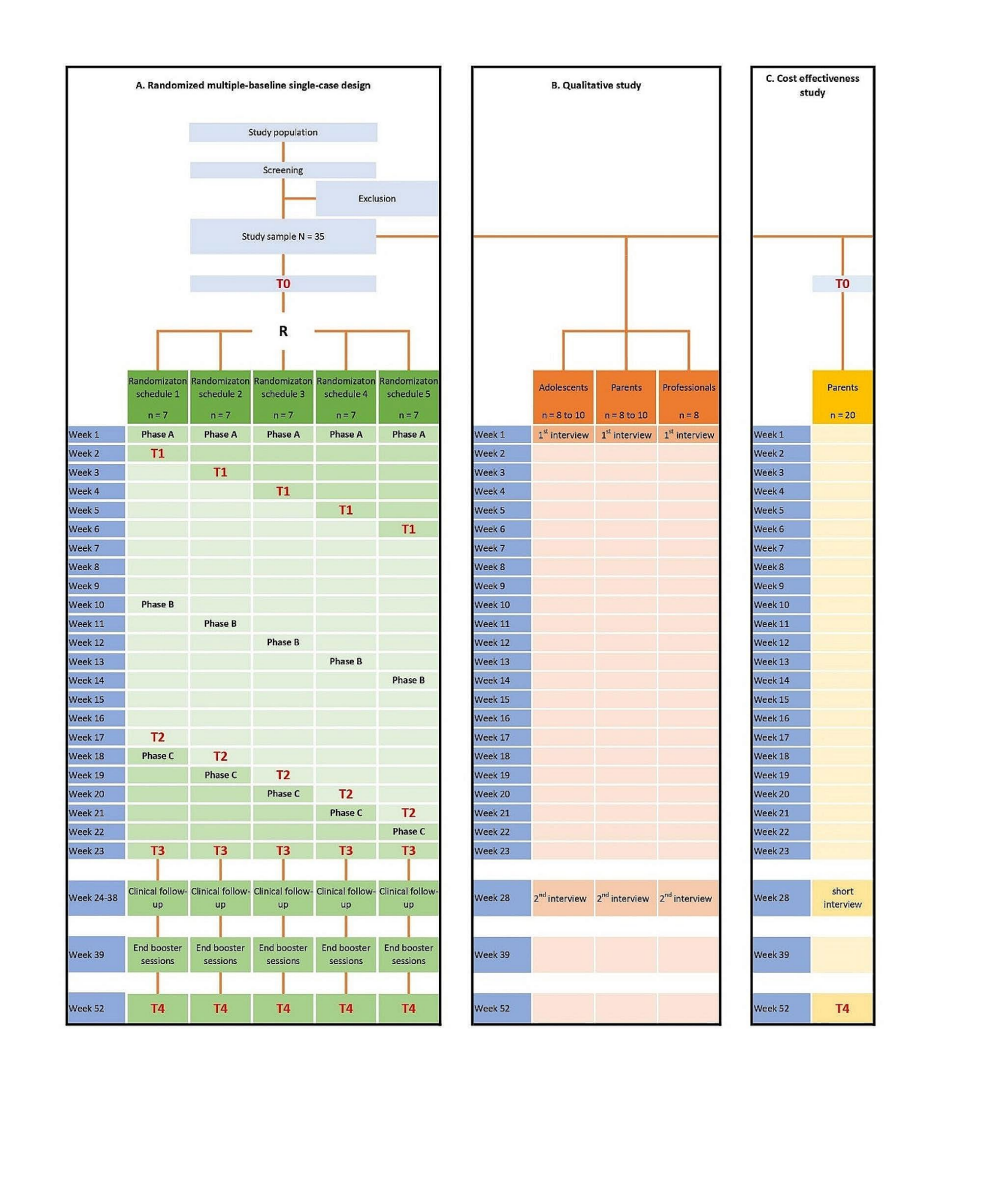
Design of the Pegasus-study

In order to be able to assess the effectiveness of the intervention, we designed a mixed-methods strategy consisting of three elements: (A) a randomized, multiple-baseline single-case design, (B) a qualitative study and (C) a cost-effectiveness study (Fig. 2).

The randomized, multiple-baseline single-case design is embedded in qualitative interviews to further evaluate the effectiveness of the intervention. Data will be collected concurrently during the project. By applying this mixed-methods design, we can integrate the results of the components and explain agreements and disagreements between the findings. Furthermore, the use of qualitative data will provide complementary information about the nature of the intervention, acceptability, perceived usefulness and barriers. This will enable us to draw more meaningful conclusions about the intervention.

### The randomized multiple-baseline single-case design

Since heterogeneity is a major aspect of the population under study, classic randomized controlled trial (RCT) models assuming homogeneity are less useful. In addition, an RCT comparing PIH with CAU would not be feasible if the treatment of choice (PIH) is withheld for a substantial amount of time from the group randomized to CAU. This group would possibly withdraw from participation immediately and/or seek PIH elsewhere, thus directly undermining the validity of an RCT. Therefore, a randomized, multiple-baseline single-case design will be conducted on 35 adolescents (11–18 years) with therapy-resistant ASD in four therapy centres.

Participants will complete repeated measurements during a baseline phase (phase A, 2–6 weeks), an intervention period (phase B, 15 weeks) and a post-intervention period (phase C, 2–6 weeks). Phase A acts as control-phase and will be compared with phase B. By applying multiple baselines of varying lengths, the observed effects of the treatment can be distinguished from effects due to chance, thus increasing internal validity. The total duration of phases A and C is 8 weeks for each participant and, consequently, participants with a longer phase A will have a shorter phase C. Participants are randomly assigned to a baseline duration by the randomization tool on the clinical data platform Castor EDC. During the total study period of 23 weeks, the parents of the participants complete an emotion regulation questionnaire (Emotion Dysregulation Inventory) three times a week to answer the primary outcome. Other assessments (ASD symptom severity, quality of life, self-esteem, global and family functioning, and goal attainment) take place at baseline (T0), at the end of baseline phase A (T1), after completion of intervention phase B (T2), after the end of post measurement phase C (T3) and after one year follow up (T4) (please see section outcomes).

#### Randomization

Participants are randomly assigned to one of five pre-defined baseline lengths to increase the internal validity of the design with a 1:1 allocation using permuted blocks of random sizes. The block sizes are disclosed to ensure concealment. Participants are randomized using the randomization tool of the clinical data platform Castor EDC. The randomization code is not released until the patient has been recruited for the trial, which will take place after the inclusion criteria have been met and an informed consent form has been signed. All patients who are willing to participate and meet the inclusion criteria are randomized.

#### Study population

Adolescents with ASD (at least 40% female or transgender to account for population diversity) are recruited within Karakter, a large child and adolescent psychiatric hospital with several branches in the Netherlands. Karakter has a large clinical outpatient programme for children and adolescents with various mental health problems, focusing particularly on neurodevelopmental disorders. Recruitment outside Karakter will be done by PIH centres Horses & Co, the Gagelhoeve and Paardenblik, as well as through social media and patient organizations (NVA and Balans). Four therapy centres have been selected to treat patients with PIH. Participants (adolescents and parents) need to be able to access the therapy centres and to sign the informed consent form and they must be willing to fill in the questionnaires. In order to be eligible, participants must comply with all of the following criteria: (1) between 11 and 18 years old; (2) a clinical diagnosis of autism spectrum disorders according to the DSM 5 as diagnosed by a BIG registered healthcare professional; (3) insufficient emotion regulation after regular therapy for at least 1.5 years, as indicated by a score above the clinical cut-off (T-score = 65) on the EDI; and (4) comorbidities are allowed except for those interfering with safety. Any potential subject who meets any of the following criteria is excluded from participation in this study: (1) unable to respond to questions (parents or adolescents); (2) no access to the Internet; (3) parents or adolescents have insufficient command of the Dutch language; (4) physically incapable of working with horses; (5) unstable medication use; (6) total IQ equal to or below 80 on the Wechsler Intelligence Scale for Children (WISC-III-R or WISC-V);7) allergic or phobic to horses; 8) insufficient regulation to safely handle the horses; and 9) had therapy with horses within the last two years.

#### Procedures

We recruit therapy-resistant and therapy-avoiding youths with ASD who are motivated to undergo PIH through social media, screening lists and screening by therapists in the participating centres. The screening procedure consists of two sessions in which patients are screened for eligibility to participation based on the inclusion and exclusion criteria. The screening is conducted by research assistants from Karakter qualified for and experienced in diagnostic assessments. Patients and their parents are informed about the intervention, aims of the study and measurements. They receive this information both orally from a research assistant and in print. As these measures have a high frequency, we place extra emphasis on the burden involved. Participants are asked to sign a written informed consent form and are referred to the equine-assisted therapists at Karakter or one of the participating centres.

After inclusion, the first assessment (T0) takes place and participants are randomly assigned to a condition. During the baseline, intervention and post-intervention phases, parents are asked to complete the EDI items three times a week by using the app M-path (https://m-path.io/landing/). This takes one to two minutes to complete. In multiple baseline design studies it is common to include multiple data points. Although the time involved in each measurement is limited, the number of measurements is substantial and therefore the burden on participants is estimated to be moderate. When there are problems with the app, they can use a questionnaire in print. For all other assessments by means of questionnaires (baseline (T0), at the end of phase A (T1), after completion of phase B (T2), after the end of phase C (T3) and after one year (T4)), we use the questionnaire platform (CASTOR EDC).

#### Descriptive and outcome measures

Outcome domains and measures and assessment frequency are shown in Table 2.

**Table 2:** Measures and frequency

Domain	Measures	Informant	Screening	T0	T1	T2	T3	T4
Descriptions	1. Intake questionnaire	Parents		X				
	2. Psychiatric diagnoses DSM-V	Professional	X					
	3. IQ screener	Adolescents	X					
Emotion Dysregulation	4. Emotion Dysregulation Inventory (EDI)	Parents	X	X	X	X	X	X
Goal attainment	5. Goal attainment scale	Adolescents			X	X		
Quality of Life	6. Kidscreen-27 - parent	Parents		X	X	X	X	X
	6. Kidscreen-27 - child	Adolescents		X	X	X	X	X
	13. Outcome Rating Scale (ORS) (each session)	Adolescents						
Broad psychopathology (internalizing and externalizing problems, thought disorders	7. CBCL/6–18	Parents		X				
	7. YRS/11–18	Adolescents		X				
	7. TRF/6–18	Teacher		X				
	7. BPM-P	Parents			X	X	X	X
	7. BPM-Y	Adolescents			X	X	X	X
	7. BPM-T	Teacher					X	
Self-esteem	8. Rosenbergh self-esteem scale (RSES)	Adolescents		X	X	X	X	X
Social functioning	9. Communication and social functioning (SRS-2)	Parents		X	X	X	X	X
	9. Communication and social functioning (SRS-2)	Teacher		X			X	
Parent-child relationship	10. Family functioning (VGFO)	Parents		X			X	
Prior beliefs	11. Prior beliefs	Parents		X				
	11. Prior beliefs	Adolescents		X				
Satisfaction	12. Satisfaction	Parents					X	
	12. Satisfaction	Adolescents					X	
	13. Session Rating Scale (SRS) (each session)	Adolescents						
Adherence	14. Intervention adherence (each session)	Professional						
Cost effectiveness	15. TiCP-Children	Parents		X				X

*Note * The lengths of phases A and C differ depending on the group the participant is randomized into

DSM-V = Diagnostic and Statistical Manual of Mental Disorders; IQ = Intelligence quotient; CBCL = Child Behaviour Checklist [6–17 YSR = Youth Self Report; TRF = Teachers report Form; BPM = Brief Problem Monitor; SRS = Social Responsiveness Scale; VGFO = Vragenlijst Gezinsfunctioneren Ouders; TiCP-Children = Trimbos and iMTA questionnaire on Cost associated with Psychiatric illness.

T1: Week before starting phase B

T2: Week before starting phase C

T3: Week after end phase C

T4: One year after start intervention

##### Intake Questionnaire (parents)

Parents are asked to report personal characteristics for assessing a range of variables about themselves and their partners, if any (gender, age, country of birth, highest completed education, family composition, living situation and occupational status) and also about the child (country of birth, medical history, medication and school parameters). This information is used to describe the included population. We administrate the intake questionnaire at T0.

##### Psychiatric diagnoses in DSM-V

At admission to one of the participating centres, information will be collected about when and by whom the diagnosis of ASD is made according to the Diagnostic and Statistical Manual of Mental Disorders, Fifth Edition (DSM-5) [31]. At Karakter, clinical DSM-5 diagnoses are established by a multidisciplinary team based on different information sources (developmental history, child observation and psychiatric assessment, and review of clinical and prior records, including information available from schools or other professional institutions involved with the child), collected by a child psychiatrist and a child psychologist, resulting in a diagnosis. Diagnoses are made by professionals with a BIG registration. For the descriptions, the primary diagnosis ASD is presented. Other diagnoses are grouped as comorbidity.

##### IQ screener

The total IQ is estimated using the Wechsler Intelligence Scale for Children (WISC-V), the most commonly used intelligence test in the Netherlands for children aged 6 to 16 years, or the Wechsler Intelligence Scale for Adults IV-NL (WAIS IV-NL), the most administrated intelligence test in the Netherlands for people aged 17 years and older [32, 33]. The WISC-V/WAIS IV-NL will only be administered if this has not been applied in the past two years. If no IQ assessment has taken place before or this is more than two years ago, TIQ will be estimated using two subtests from the WISC-V/WAIS IV-NL (block patterns, vocabulary). This will be done in the screening phase.

##### Emotional dysregulation (primary outcome)

The primary outcome is emotional dysregulation as measured by the Emotion Dysregulation Inventory (EDI) [34, 35]. The Emotion Dysregulation Inventory (EDI) is specifically designed to measure emotion regulation impairments in youths and adolescents with ASD. The EDI-Short Form is a validated, change-sensitive, 13-item caregiver report measure of emotion regulation impairment for individuals who are at least 6 years of age. The EDI was developed based on the item response theory (IRT) analysis and none of the final items had evidence of differential item functioning (e.g., psychometric biases) by gender, age, intellectual ability and verbal ability, making it suitable for use across heterogeneous populations. Items on the EDI measure how problematic behaviours have been during the past day. The scale used is Not at all = 0, Mild = 1, Moderate = 2, Severe = 3, or Very Severe = 4. The EDI short form includes two scales: a 7-item Reactivity Index and a 6-item Dysphoria Index. Index raw scores can be converted into t-scores or theta scores based on a sample of 1755 individuals with ASD [35] or on a sample of 1000 youths matching the US census as general population norms [34]. For the purposes of this study, we administered the 13-item EDI short form at T0, three times a week (for 23 weeks) during a baseline phase (phase A, 2–6 weeks), an intervention period (phase B, 15 weeks) and a post-intervention period (phase C, 2–6 weeks), and once at T4. We will ask parents to rate the last two days.

##### Goal Attainment Scale

The Goal Attainment Scale (GAS) [36] is a method of scoring to what extent the patient’s individual goals are achieved in the course of the intervention and is part of the intervention as well. Each patient has his own outcome measure, but this is measured in a standardized way to allow statistical analysis. Each goal is rated on a 6-point scale, capturing the degree of attainment in each goal area. In the three system sessions we visualize the GAS with six pylons in a row and the participants are invited to walk from the pylon representing the situation at the start to the pylon representing the current situation. This can be worse, the same as at the start, little improvement, satisfied, better than expected or much better than expected. Then the parents are invited to do the same. Scores are recorded on a score form. The initial situation at start is scored − 2 and a little improvement is scored − 1. When the patient achieves the intended level, this is scored 0. When he achieves a better than expected outcome, this is scored + 1 (more than target) or + 2 (much more than target). Achieving a worse than expected outcome is scored − 3 (decline). In this study, a maximum of three goals are identified, which are incorporated into a single GAS score. We adjusted the GAS to suit the patient group. As they often take words literally, we did not want them to be confronted with a score of 0 to prevent them from thinking, “I am a null”. Therefore, the patients do not see their scores. The GAS is assessed from participants and parents in sessions number six, ten and fifteen.

##### Kidscreen-27 (parents) and Kidscreen-27 (adolescents)

The KIDSCREEN-27 (https://www.kidscreen.org/english/questionnaires/kidscreen-27-short-version/) [37] is a generic health-related quality of life (HRQOL) questionnaire for children and adolescents applicable to healthy and chronically ill children and adolescents aged between 8 and 18 years. There are two versions of the questionnaire, being a self-completed version (child/adolescent) and a proxy version (parent/proxy). The KIDSCREEN-27 consists of 27 items and measures five dimensions: physical well-being, psychological well-being, parent relations & autonomy, social support & peers, and school environment. The recall is one week. Children and parents rate items on a 5-point Likert-type scale assessing frequency (never [1], seldom [2], sometimes [3], often [4] and always [5]) or intensity (not at all [1], slightly [2], moderately [3], very [4] and extremely [5]). Scores are coded from 1 to 5. Negatively formulated items are recoded and the sum scores for respective dimensions are converted into T scores with a mean of 50 and a standard deviation (SD) of 10. Higher scores match with a better HRQOL. The KIDSCREEN-27 has good psychometric properties. The internal consistency of the domains is between 0.81 and 0.84, and the test-retest reliability of the domains ranges from 0.61 to 0.74. The KIDSCREEN-27 (parents and adolescents) is assessed at baseline (T0), at the end of phase A (T1), after completion of phase B (T2), after the end of phase C (T3) and after one year (T4).

##### Global functioning (CBCL/6–18, YRS/11–18, TRF/6–18)

To assess emotional and/or behavioural problems, we use the CBCL/6–18 (https://aseba.org/) [38], completed by parents or substitutes, the TRF/6–18, completed by teachers and other school staff, and the YSR/11–18, completed by the youths. All three questionnaires include more than 100 items assessing behavioural and emotional problems that are answered on a 3-point Likert-type scale (0 = not true, 1 = somewhat or sometimes true, 2 = very true or often true) by parents. The scores display eight problem scales (withdrawn [1]; somatic [2]; anxious [3]; social [4]; thought [5]; attention [6]; rule-breaking [7]; aggressive [8]) and other problems. The ‘internalizing behaviour’ scale is formed by the sum of the problem scales 1, 2 and 3, and the ‘externalizing behaviour’ scale by the sum of problem scales 7 and 8. The total problem scale is formed by all subscales together. Some items contribute to more than one problem scale. T-scores are computed from raw scores; higher scores on the syndrome scales indicate more problems. T-scores of 63 (90th percentile) indicate the clinical range, indicating professional help is needed. For the competence scales, lower scores indicate greater severity. A T-score < 37 indicates the clinical range. The CBCL/6–18 has robust psychometric properties in clinical, non-clinical and cross-cultural populations. The CBCL 6–18 (parents), YSR (adolescents) and TRF (teachers) are assessed at baseline (T0).

The Brief Problem Monitor (BPM) (https://aseba.org/) [39] is a rating instrument for monitoring children’s functioning and responses to interventions and a short version of the CBCL/YSR/TRF. The BPM consists of a minimum set of items that can be completed in 1 to 2 min by parents (BPM-P), teachers (BPM-T) and youths (BPM-Y). 19 items of the CBCL/6–18 (or TRF or YSR) make up the BPM: 6 items for attention (all 6 from the CBCL/6–18 attention problems scale), 7 items for externalizing (all 7 from the CBCL/6–18 aggression scale) and 6 items for internalizing (5 from the CBCL/6–18 anxiety/depression and 1 from the CBCL/6–18 withdrawn/depressed scales). Each item is rated as 0 = not true, 1 = somewhat true, or 2 = very true. Parallel items and scales on the BPM & the CBCL/6–18, TRF and YSR enable us to link comprehensive initial and outcome assessments with BPM scores. The BPM-P and BPM-Y are assessed at the end of phase A (T1), after the completion of phase B (T2), after the end of phase C (T3) and after one year (T4). The BPM-T (teachers) is assessed at the end of phase C (T3).

##### Rosenbergh self-esteem Scale (RSES)

The Rosenberg Self-Esteem Scale (RSES) [40] is used to assess self-esteem. It is a widely used 10-item Likert-scale self-esteem measure. Items are answered on a 4-point scale — ranging from strongly agree to strongly disagree — measuring positive and negative feelings towards the self. The Dutch version of the RSES is found to be a one-dimensional scale with high internal consistency and congruent validity and a Cronbach’s alpha of 0.89 [40]. The Rosenberg Self-Esteem Scale is assessed at baseline (T0), end of phase A (T1), after phase B (T2), after phase C (T3) and after one year (T4).

##### Social Responsiveness Scale – Second edition (SRS-2)

The Social Responsiveness Scale – Second edition (SRS-2) [41] measures deficits in social behaviour associated with ASD and can be used to assess the severity of symptoms in ASD [42]. The questionnaire is completed by multiple raters (parents and teachers). The SRS-2 consists of 65 items scored on a Likert scale ranging from not true = 1, sometimes true = 2, often true = 3 to almost always true = 4. It is designed to identify social impairments intrinsic to ASD and to quantify its severity across the duration of the treatment. A raw total score and five treatment subscale scores (Social Awareness; Social Cognition; Social Communication; Social Motivation; Restricted Interests, and Repetitive Behaviour) are obtained. Raw total scores are converted into gender-normed *T* scores. The accepted diagnostic criteria (cut-off point) for the SRS-2 with a diagnosis of ASD are: *T* scores < = 59 (normal); *T* scores 60–75 (mild to moderate ASD); and *T* scores = > 76 (severe ASD). The SRS-2 (parents) is assessed at baseline (T0), at the end of phase A (T1), after completion of phase B (T2), after the end of phase C (T3) and after one year (T4). The SRS-2 (teachers) is assessed at baseline (T0) and at the end of phase C (T3). The SRS-2 (parent report, Dutch version) demonstrates high internal consistency (Cronbach’s alpha ranged from 0.92 to 0.95, good convergent validity (*r* = .63 with the ADI-R) and is able to differentiate between children with ASD and the general population [43].

##### Family Functioning Questionnaire (VGFO, 34 items)

The Family Functioning Questionnaire [44] can be answered on a 4-point scale ranging from 1 (not applicable) to 4 (completely applicable) with lower scores indicating more problems with family functioning. There are five subscale scores (basic care, parenting, social contacts, youth experience, and partner relationship). Together, they form the total score of the VGFO. A percentile score and T-score are calculated for each subscale and for the total. Based on the T-scores, the parent’s functioning can be interpreted as having no significant problems, mild problems or severe problems. The VGFO demonstrated high internal consistency for the subscales (Cronbach’s alpha ranges from 0.70 to 0.90). For the total scale the alpha is 0.90 [45].This questionnaire is assessed at baseline (T0) and at the end of phase C (T3).

##### Prior beliefs and motivation for therapy

Prior beliefs of parents and adolescents about the short-term and long-term success and burden of the intervention are evaluated using a 6-item questionnaire in which parents and adolescents rate their prior beliefs on a 10-point scale ranging from 1 (completely disagree) to 10 (completely agree). This measurement will also be applied to examine if randomization is successful. This will be done by verification if the five groups do not differ in beliefs about the effectiveness of the intervention at baseline. Prior beliefs of parents and adolescents are assessed at baseline (T0).

##### Satisfaction

Satisfaction is measured at the end of phase C (T3) using a self-constructed 6-item questionnaire in which parents and adolescents rate their satisfaction on a 10-point scale ranging from 1 (completely disagree) to 10 (completely agree).

##### SRS/ORS

The Outcome Rating Scale (ORS) and Session Rating Scale (SRS) is used to assess the well-being of the child and the way the patient experiences the intervention (https://www.scottdmiller.com/scholarly-publications-handouts-vitae/) [46]. At the start of each session, the child and the parents (if attending the session) complete the Dutch translation of the Outcome Rating Scale (ORS) on paper by putting a mark on a visual 10-centimetre scale, ranging from ‘very bad’ on the left side to ‘very good’ on the right side. The ORS consists of four items about the well-being of the child [at [1] individual level [2], family level [3] social level and [4] general level]. Similarly, at the end of each session, the child completes the Dutch translation of the Session Rating Scale (SRS) for themselves, which also contains four items about the way they perceived the session [ [1] relationship [2], goals and setting [3], approach and methods, and [4] overall]. The SRS allows treatment sessions to be evaluated at any time to ascertain whether or not treatment is ‘on the right track’ toward a successful outcome [47]. Parents rate their own well-being and experience with the intervention during the system sessions. Research has shown that the translated Dutch ORS and SRS have sufficient reliability and a limited validity for the Dutch adult [48]. The Dutch ORS and SRS are suitable questionnaires for following progress during treatment, but the use of a second questionnaire for measuring treatment outcome is recommended [49]. So far, only one study has reported on the psychometric properties of the ORS for children. This demonstrates that the ORS and the CORS display strong evidence of reliability with coefficient alpha estimates of 0.93 and 0.84 respectively [50].

##### Adherence

Fifteen sessions are scheduled. Cancelled sessions are rescheduled until all sessions have taken place. Dropouts with reason for this are noted.

##### TiCP-Children

The Trimbos and iMTA questionnaire on Cost associated with Psychiatric illness (TiCP-Children) is a questionnaire developed to evaluate the economic value of interventions for children with psychological problems [51]. It consists of three parts. The first part asks for demographic information of the child and the reporting parent. The second part (questions 11–39) concerns medicine use and the last part contains questions about school dropout and lost free time activities. Optional questions can be added beforehand, depending on the research population. The recall period is 3 months. The TiCP-Children are completed at T0 and T4.

#### Data collection and management

All participants receive a unique code assigned at inclusion to anonymize all data. Informed consents, outcomes of IQ subtests and written questionnaires, such as the ORS and SRS, are stored in a locker in a locked archive. Only appointed researchers have access to the archive and the locker. Other data are stored in CASTOR, on-line electronic data capture software. Research assistants check the information on a weekly basis for completeness and contact parents by e-mail or telephone when questionnaires have not been completed on time. At the end of the study, parents and participants receive 50 euro and the travel costs to the locations of the intervention.

#### Sample size

The tool https://architecta.shinyapps.io/power_MBD/ was used to give an estimate of the power using the following input: the number of participants is 30, the number of measurements is 69 (three times a week for 23 weeks), the number of possible starting points is 5, the minimum number of baseline measurements is 8 (once a week; phases A + C), the minimum number of intervention measurements is 15 (once a week; phase B), the proportion of baseline measurements is 35%, the proportion of overlapping starting moments is 80%, the effect size is 0.6, the type 1 error rate (alpha) is 0.05, the autocorrelation is 0.15 and the number of emerging effect measures is 3. In order to be able to do the power simulations, we specified the number of samples that have to be drawn to calculate the power (150 samples) and the number of times this power has to be simulated [5]. Using the above parameters we found a power of 0.83. To account for possible drop-outs, we added another five participants, thus a total of 35 participants are included. For the secondary outcomes we will correct for multiple testing by the False Discovery Rate (FDR) [52].

#### Data analyses

Data will be analysed using SPSS V.24.0 for Windows (SPSS Incorporated). Descriptive statistics will be performed for baseline characteristics of the study population. Parametric data will be presented as means with standard deviation (SD) and non-parametric distributed variables as median with interquartile ranges (IQRs). For the primary outcome we will combine visual analyses, randomization tests and effect sizes for a comprehensive analysis of the intervention based on state-of-the-art knowledge on analysing a randomized multiple-baseline single-case design [53].

##### Time series analyses

Firstly, data will be visually inspected within and between phases with respect to (1) level (2) trend (3) variability (4) immediacy of the effect (5) overlap and (6) consistency of data across similar phases [35, 36]. Secondly, the intervention effect will be statistically tested by applying a sequential approach. We will test the null hypothesis of no treatment effect for any of the cases with the non-parametric approach to combine randomization test p-values [54]. If it is determined that the intervention has indeed a statistically significant effect on emotion regulation, we will use the hierarchical linear modelling [38] approach for analysing the data in closer detail in order to obtain the parameter estimate of and to further model the average treatment and individual treatment effects. The average treatment effect is an effect size and will as such be compared with the meta-analytic effect size as obtained in other studies. No statistical testing will be performed in the final step. The interaction between treatment effect and time will be included in the hierarchical linear model in order to model differential trends.

##### Single time points analyses

Pre-treatment scores on the EDI will be compared with post-treatment and follow-up scores on the EDI and other secondary measurements using a non-parametric Wilcoxon signed rank test. An effect size will be obtained from this analysis with the follow-up versus pre-treatment comparison as input for this effect size.

### Qualitative study

The aim is to obtain information from a varied group of adolescents, parents and professionals to gain insight into their individual experiences as well as the effects of the intervention.

#### Participants

Purposive sampling identifies those participants who are most likely to provide rich information about their experiences. The study population consists of three groups:

1. Patients referred to the intervention (*n* = 8–10).
2. Parents of the adolescents referred to the intervention (*n* = 8–10).
3. The professionals delivering the intervention (*n* = 8).

We use a maximum variation sampling approach to purposefully select a wide range of participants [39]. The sample size particularly depends on the complexity of the research and the heterogeneity of the relevant characteristics. However, it is expected that 8–10 participants will be included in each group (patients and parents). The final number of subjects, however, will be determined on the basis of saturation. We will interview all eight members of the professionals group.

#### Procedure

The first contact of patients and parents is the research assistant. She introduces the qualitative researcher. Parents and children receive a formal letter explaining the qualitative part of the study and an informed consent form. Only those who have given their written informed consent to schedule an interview are invited to be interviewed. All interviews are assessed by one or two qualitative researchers. Interviews take 45–60 min. Parents and adolescents are interviewed separately. Each participant is interviewed twice (before the start of the intervention and 4–6 weeks after the intervention).

The professionals are interviewed at their workplace. All interviews are assessed by a qualitative researcher and an experienced mental health professional. Interviews last for 45–60 min. Each professional is interviewed twice (at the start of the project and when all participants are included).

#### Data collection

A total of *n* = 8–10 adolescents and their parents are invited to participate in individual semi-structured in-depth interviews of one hour, preferably face to face or, if this is not possible, by video call. The interviewers ask open questions in order to obtain a complete picture of the participants’ experiences [55]. An iterative topic list is used to explore the knowledge and experiences about the intervention. Possible examples of questions for adolescents and their parents include: What do you expect from the PIH intervention? What are the facilitators and barriers? What benefits have you derived from the PIH intervention? What should be maintained in the intervention? How did you experience the sessions? How did you experience the therapist? Etc.

PIH therapists are interviewed to describe and evaluate therapeutic elements and implementation processes. Possible examples of questions are: What characterizes the PIH intervention? What are the facilitators and barriers for referral to PIH? How do participants perceive the PIH? What aspects are missing from your perspective? How is the intervention implementation experienced by PIH therapists? Etc.

#### Data analysis

The interviewers audiotape the interview with the participant’s permission. After the interview the participants’ nonverbal behaviours and emotions are logged. A reflexive journal will record the overall process of data collection. The data collection and analysis occur concurrently. After each interview, the recording is literally transcribed and coded to reveal broad or initial categories or themes. This process is based on Grounded Theory and will exist of open coding, followed by axial coding and selective coding [56]. Interview transcripts and observation narratives are coded by two researchers.

ATLAS-ti 9, a software package for qualitative research, is used for coding, management and data retrieval. During the analysis, researchers use memos and diaries to reflect on the data and discuss themes over and over again. We compare interview data from adolescents and parents to examine the intervention from different perspectives.

On completion of the study, the data will be analysed and a final study report will be written. Participants will be notified of the outcome of the study, either by provision of the publication or via another form of communication.

### C. cost effectiveness study

We will use the Trimbos /iMTA Questionnaire for Costs associated with Psychiatric Illness (TiCP-Children) as well as interviews with parents to answer questions on cost-evaluation and the effect of improved well-being. Using the TiCP-Children and the interviews we will be able to analyse the social benefits of PIH and the costs saved by society due to positive results of PIH.

#### Participants

All parents of the participants are asked to complete the TiCP-Children and all parents who have not participated in the qualitative interviews are asked to share their experiences with the investigators using a semi-structured interview. The interviews will take place approximately four to six weeks after the intervention has been finished.

#### Study procedure

The TiCP-Children is completed on T0 and T4 together with the other questionnaires at T0 and T4. Parents (*n* = 20) who have not participated in the qualitative interviews are asked to participate. Written informed consent is required. The interviews will be held by video call and take approximately 30–60 min.

#### Data collection

Two questions are central in the interviews: What are the barriers and what the benefits of the PIH intervention?

Five of the more reflective questions will be presented to those involved.

1. Please rate: How do you rate the intervention?
2. Benefits: Which improvements are made by the intervention?
3. Facilitators: What made it easy to participate in the intervention?
4. Thresholds: What made it difficult to participate in the intervention?
5. Points for improvement: Do you have any suggestions for improving the intervention?

#### Data analysis

To calculate the costs of the medical consumption the number of contacts is multiplied by the costs per consult. We will use reference values to estimate the costs per consults. The outcomes of the interviews will be described in terms of social benefits (such as back to school, less reactivity and improved emotion regulation, less violent/angry, etc.), facilitators and barriers. The result will be an overview of the effectiveness of the intervention.

## Discussion

The goal of the Pegasus project is to quantify the short-term (15 weeks) and long-term (1 year) (cost)effectiveness of Psychotherapy incorporating horses (PIH) in adolescents with therapy-resistant ASD (aged 11–18) and, when proven (cost)effective, implement PIH in clinical practice.

In this paper we describe the rationale, study design and methods of this ongoing project.

Several studies examined the effects of equine-assisted interventions in children with ASD, although these had several methodological limitations. As far as we know, this is the first study [1] examining the effect of this form of PIH (one therapist, unmounted activities with or in the presence of the horse) [2], in this particular group (adolescents with ASD and ongoing emotion regulation problems after at least 1.5 year of therapy) and [3] combining a quantitative, qualitative and cost-effectiveness study on the effect of PIH.

Using a mixed method design gives us the opportunity to interpret our results in a broader scope. Results from static questionnaires do not always catch subtle changes, whereas we can only expect little steps forward in this severely affected group.

A possible pitfall of our study is therapy tiredness of both children and parents, which may result in a high drop-out level. The combination of a weekly therapy session with participation in this study may be too demanding for these highly vulnerable families. Another pitfall may be related to the fact that four centres are participating, which may hamper the consistency of the intervention. Of course, this is mended through a rigorous protocol and intervision sessions. We include a highly affected group of adolescents with ASD, so there can be no generalization of results. However, any positive effect in this special group will be promising for other groups. Data collection will end one year after the last inclusion.

## Data Availability

No datasets were generated or analysed during the current study.

## Abbreviations

ASD: Autism spectrum disorder
BIG: Beroepen Individuele Gezondheidszorg
BPM: Brief Problem Monitor
BWP: Belgian Warmblood Paard
CAU: Care as usual
CBCL: Child Behaviour Checklist
CBT: Cognitive Behaviour Therapy
DSM-V: Diagnostic and Statistical Manual of Mental Disorders
EAT: Equine-assisted therapy
ED: Emotion dysregulation
EDI: Emotion Dysregulation Inventory
IQRs: Interquartile ranges
IQ: Intelligence quotient
FDR: False Discovery Rate
GAS: Goal Attainment Scale
KWPN: Koninklijk Warmbloed Paard Nederland
NVA: Nederlandse Vereniging Autisme
PIH: Psychotherapy Incorporating Horses
RCT: Randomized Controlled Ttrial
RM: Relationship management
RSES: Rosenbergh self-esteem scale
SD: Standard Deviation
SoA: Social awareness
SeA: Self-awareness
SM: Self-management
SKJ: Dutch independent register for youth professionals
SRS: Social Responsiveness Scale
TiCP: Trimbos and iMTA questionnaire on Cost associated with Psychiatric illness
TRF: Teachers Report Form
VGFO: Vragenlijst Gezinsfunctioneren Ouders
WAIS: Wechsler Intelligence Scale for Adults

## References

[1] Fombonne E, Editorial (2018). The rising prevalence of autism. J Child Psychol Psychiatry Allied Discip.

[2] Aldao A, Nolen-Hoeksema S, Schweizer S (2010). Emotion-regulation strategies across psychopathology: a meta-analytic review. Clin Psychol Rev.

[3] Samson AC, Wells WM, Phillips JM, Hardan AY, Gross JJ (2015). Emotion regulation in autism spectrum disorder: evidence from parent interviews and children’s daily diaries. J Child Psychol Psychiatry Allied Discip.

[4] Nader-Grosbois N, Mazzone S (2014). Emotion regulation, personality and Social Adjustment in Children with Autism Spectrum disorders. Psychology.

[5] Swain D, Scarpa A, White S, Laugeson E (2015). Emotion dysregulation and anxiety in adults with ASD: does Social Motivation play a role?. J Autism Dev Disord.

[6] Mazefsky CA, Borue X, Day TN, Minshew NJ (2014). Emotion regulation patterns in adolescents with high-functioning autism spectrum disorder: comparison to typically developing adolescents and association with psychiatric symptoms. Autism Research: Official J Int Soc Autism Res.

[7] Mazefsky CA (2015). Emotion regulation and emotional distress in Autism Spectrum Disorder: foundations and considerations for Future Research. J Autism Dev Disord.

[8] Mogensen L, Mason J (2015). The meaning of a label for teenagers negotiating identity: experiences with autism spectrum disorder. Sociol Health Illn.

[9] Gillespie-Lynch K, Kapp SK, Brooks PJ, Pickens J, Schwartzman B. Whose expertise is it? Evidence for autistic adults as critical autism experts. Front Psychol 2017;8(438). 10.3389/fpsyg.2017.00438 PMC5368186 28400742

[10] Fuller EA, Kaiser AP (2020). The effects of Early Intervention on Social Communication Outcomes for Children with Autism Spectrum disorder: a Meta-analysis. J Autism Dev Disord.

[11] Reichow B (2012). Overview of meta-analyses on early intensive behavioral intervention for young children with autism spectrum disorders. J Autism Dev Disord.

[12] Peters-Scheffer N, Didden R, Korzilius H, Sturmey P (2011). A meta-analytic study on the effectiveness of comprehensive ABA-based early intervention programs for children with Autism Spectrum disorders. Res Autism Spectr Disorders.

[13] Malter Cohen M, Tottenham N, Casey BJ (2013). Translational developmental studies of stress on brain and behavior: implications for adolescent mental health and illness?. Neuroscience.

[14] Mengler L, Khmelinskii A, Diedenhofen M, Po C, Staring M, Lelieveldt BP (2014). Brain maturation of the adolescent rat cortex and striatum: changes in volume and myelination. NeuroImage.

[15] Burnett Heyes S, Jih YR, Block P, Hiu CF, Holmes EA, Lau JY (2015). Relationship reciprocation modulates Resource Allocation in Adolescent Social networks: Developmental effects. Child Dev.

[16] Sussman D, Leung RC, Vogan VM, Lee W, Trelle S, Lin S (2015). The autism puzzle: diffuse but not pervasive neuroanatomical abnormalities in children with ASD. NeuroImage Clin.

[17] Fein D, Barton M, Eigsti IM, Kelley E, Naigles L, Schultz RT (2013). Optimal outcome in individuals with a history of autism. J Child Psychol Psychiatry Allied Discip.

[18] McDaniel Peters BC, Wood W (2017). Autism and equine-assisted interventions: a systematic mapping review. J Autism Dev Disord.

[19] Trzmiel T, Purandare B, Michalak M, Zasadzka E, Pawlaczyk M (2019). Equine assisted activities and therapies in children with autism spectrum disorder: a systematic review and a meta-analysis. Complement Ther Med.

[20] Nieforth LO, Schwichtenberg AJ, O’Haire ME (2023). Animal-assisted interventions for Autism Spectrum disorder: a systematic review of the literature from 2016 to 2020. Rev J Autism Dev Disorders.

[21] Wood W, Alm K, Benjamin J, Thomas L, Anderson D, Pohl L (2021). Optimal Terminology for Services in the United States that incorporate horses to Benefit people: a Consensus Document. J Altern Complement Med (New York NY).

[22] Goodman G, Calderon A, Midgley N (2022). Expert clinicians’ prototypes of an adolescent treatment: common and unique factors among four treatment models. Psychother Res.

[23] Midgley N, Ensink K, Lindqvist K, Malberg N, Muller N. Mentalization-based treatment for children: a time-limited approach. American Psychological Association.; 2017.

[24] Weijers JG, ten Kate C, Debbané M, Bateman AW, de Jong S, Selten JPCJ (2020). Mentalization and psychosis: a rationale for the Use of Mentalization Theory to understand and treat non-affective psychotic disorder. J Contemp Psychother.

[25] Goepfert E, Mulé C, von Hahn E, Visco Z, Siegel M (2015). Family System interventions for families of children with Autism Spectrum Disorder. Child Adolesc Psychiatr Clin N Am.

[26] Meijer MaW R. Geen woorden maar paarden, equine assisted coaching & therapie2023. 246 p.

[27] Srinivasan SM, Cavagnino DT, Bhat AN (2018). Effects of Equine Therapy on individuals with Autism Spectrum disorder: a systematic review. Rev J Autism Dev Disord.

[28] Malinowski K, Yee C, Tevlin JM, Birks EK, Durando MM, Pournajafi-Nazarloo H (2018). The effects of equine assisted therapy on plasma cortisol and oxytocin concentrations and heart rate variability in horses and measures of symptoms of post-traumatic stress disorder in Veterans. J Equine Veterinary Sci.

[29] Dobson D DK. Evidence-based practice of cognitive-behavioral therapy: Guilford publications; 2018.

[30] Dobson D, Dobson KS. Evidence-based practice of cognitive-behavioral therapy, 2nd ed. New York, NY, US: Guilford Press; 2017. xiii, 354-xiii, p.

[31] American Psychiatric Association (2013). Diagnostic and statistical manual of mental disorders: DSM-5.

[32] Wechsler D, Hendriks MPH, Ruiter S, Schittekatte M, Bos A (2018). WISC-V-NL: Wechsler Intelligence Scale for children :Nederlandstalige bewerking. Tweede druk ed.

[33] Wechsler D, WAIS-IV-NL (2018). Wechsler adult intelligence scale-fourth edition-nederlandstalige bewerking. Tweede druk.

[34] Mazefsky CA, Yu L, Pilkonis PA. Psychometric properties of the emotion dysregulation inventory in a nationally Representative Sample of Youth. J Clin Child Adolesc Psychol. 2020:1–13. 10.1080/15374416.2019.1703710 PMC7781089 31910035

[35] Mazefsky CA, Day TN, Siegel M, White SW, Yu L, Pilkonis PA (2018). Development of the emotion dysregulation inventory: a PROMIS(R)ing method for creating sensitive and unbiased questionnaires for Autism Spectrum Disorder. J Autism Dev Disord.

[36] Kiresuk TJ, Sherman RE (1968). Goal attainment scaling: a general method for evaluating comprehensive community mental health programs. Commun Ment Health J.

[37] The KIDSCREEN Group Europe (2006). The KIDSCREEN questionnaires - quality of life questionnaires for children and adolescents. Handbook.

[38] Verhulst FC, Van der Ende J (2013). Handleiding ASEBA-Vragenlijsten voor leeftijden 6 t/m 18 jaar: CBCL/6–18, YSR en TRF.

[39] Achenbach TM, McConaughy SH, Ivanova MY, Rescorla LA (2011). Manual for the ASEBA brief Problem Monitor™ (BPM).

[40] Franck E, De Raedt R, Barbez C, Rosseel Y (2008). Psychometric properties of the Dutch Rosenberg Self-Esteem Scale. Physiol Belgica.

[41] Constantino JN, Davis SA, Todd RD, Schindler MK, Gross MM, Brophy SL (2003). Validation of a brief quantitative measure of autistic traits: comparison of the social responsiveness scale with the autism diagnostic interview-revised. J Autism Dev Disord.

[42] Frazier TW, Youngstrom EA, Speer L, Embacher R, Law P, Constantino J (2012). Validation of proposed DSM-5 criteria for autism spectrum disorder. J Am Acad Child Adolesc Psychiatry.

[43] Roeyers HTM, Druart C, De Schryver M, Schittekate M (2011). SRS Screeningslijst voor autismespectrumstoornissen. Handleiding.

[44] Veerman JW, Kroes G, De Meyer R, Janssen J, Nguyen L, Vermulst A (2016). Handleiding VGFO. Vragenlijst Gezinsfunctioneren volgens Ouders.

[45] Veerman JW, Kroes G, De Meyer R, Janssen J, Janssen J, Nguyen L, Vermulst A, Manual VGFO (2016). Questionnaire on Family Functioning according to parents.

[46] Miller SD, Duncan BL (2004). The outcome and session rating scale. Administration and scoring manual.

[47] Melissa G, Murphy SRRMH. The Psychometric properties of the Session Rating Scale: a narrative review. J Evid Based Soc Work.17:3279–99. 10.1080/26408066.2020.1729281 32420833

[48] Hafkenscheid ADB, Miller SD. The outcome and session rating scales: a cross-cultural examination of the psychometric properties of the Dutch translation. J Br Ther (7):1–12.

[49] Janse P, Boezen-Hilberdink L, van Dijk MK, Verbraak MJPM, Hutschemaekers GJM (2014). Measuring feedback from clients. Eur J Psychol Assess.

[50] Duncan BLSJ, Miller SD et al. l. Giving youth a voice: a preliminary study of the reliability and validity of a brief outcome measure for children, adolescents, and caretakers. J Br Ther 5:71–87.

[51] Straten LR, Tiemens A, Donker B. M. Manual Trimbos/iMTA Questionnaire for Costs Associated with Psychiatric Illness (TIC-P) (in Dutch). 2002;2.

[52] Benjamini Y, Yekutieli D (2005). False Discovery rate–adjusted multiple confidence intervals for selected parameters. J Am Stat Assoc.

[53] Heyvaert M, Moeyaert M, Verkempynck P, Van den Noortgate W, Vervloet M, Ugille M (2017). Testing the intervention effect in single-case experiments: a Monte Carlo Simulation Study. J Exp Educ.

[54] Manly BFJ. Randomization Tests, 4th Edition by, Edgington ES. Patrick Onghena. Int Stat Rev. 2007;75(2):269-.

[55] Kvale S, Brinkmann S (2009). Interviews: learning the craft of qualitative research interviewing.

[56] Baarda BB. E ea. Basisboek Kwalitatief onderzoek, Handleiding voor het opzetten en uitvoeren van kwalitatief onderzoek. 4e druk ed2018. 336 p.

